# Supplementary material for: Self-organized division of cognitive labor
Source: PLoS One. 2021 Jul 19;16(7):e0254532. doi: 10.1371/journal.pone.0254532 (PMC8289079; doi:10.1371/journal.pone.0254532)
Supplement: Attachment — Submitted filename: Response to Reviewers.docx [file pone.0254532.s001.docx]

**Response to Reviewers**

Response to academic editor’s points:

- Laboratory protocols were suggested to be deposited in protocols.io. Our protocols were uploaded to said repository and the link was added to the ‘Materials and methods’ section in the paper.
- Additional details regarding participant consent were requested. The ‘Participants and procedure’ section in the manuscript was augmented to make explicit that informed consent was requested from participants before they could proceed to the test and that no minors were involved in it.
- The grant information was fixed to match ‘Funding Information’ and the financial disclosure.

Response to reviewer 1’s comments:

- The abstract now contains an indication of the main result.
- We added to the ‘Discussion’ section some suggestions on how our findings are applicable to human-robot collaboration and multi-agent systems, and a paragraph in the ‘The task’ section to show how we can model some real-world situations.
- The reviewer rightly pointed out that our claim that game-theoretic explanations provide only distal causes of behavior is too strong. We have relaxed our statement by appealing to Lieder’s and Griffiths’ “Resource-rational analysis” paradigm. See the ‘Introduction’ section for more details.
- Relaxing the abovementioned claim also addresses the reviewer’s point that our experiment cannot say anything about maximization of utility being relevant or not, since our subjects did not receive incentives for their performance.
- The Participants and procedure section has been updated with a more complete description of the students that participated in the experiment, who were NOT from either of our classes, but from the standing human subject pool at Indiana University.
- The reviewer raised an issue with the regressions in Figure 5 top left and Figure 7 top right. Both of these regressions feature scatter plots of consistency on round n vs. score on round n-1 (the former for behavioral data and the latter for simulated data). It is correct to claim that a linear model is not the right model to fit in this case. For one thing, the points are too far away from the means, there is heteroscedasticity and too much variance. However, we are not fitting a model for predictive purposes. The figures are just intended to give a visual way to present a tendency in the qualitative behavior. Tendencies – viz Win Stay, Lose Shift – can be appreciated from the respective Pearson correlations, which correspond to the slants of the linear regressions. We included “hedging” phrases in the manuscript to warn the reader about this particular use of linear regressions and included the Pearson correlations. We would also like to point out that a logistic regression is no better an option than linear regression here because the outcome variable (consistency on round n) takes ordered values.
- As for the interaction coefficient in the regression DLindex ~ Consistency + DifConsistency*Overlap, the reviewer pointed out that the result might be different if Consistency was not included. However, the interaction coefficient is even higher and significant when this contribution is excluded ($\beta_{3}\approx0.021;p-value<0.001$; observe that the interaction coefficient is now $\beta_{3}$, not $\beta_{4}$). But we think that the effect of consistency should be taken into consideration to isolate the interaction effect, since consistency already has quite an important contribution to DLindex. We added a phrase in the manuscript to point out that the sign of the contribution doesn’t change if we leave out the contribution of consistency.
- Concerning the question of how players react to the unicorn being present or not, we have modified the second paragraph in the ‘Results’ section to address this question. We show that rounds with unicorn present made players more active, in the sense that they explored a larger portion of the grid on the next round.
- The typo on page 10 and the formatting error in the references were corrected.

Response to reviewer 2’s comments:

- The reviewer rightly points out that the task is quite complicated. This is a point to be taken into account for future experiments. However, we justify our task on the basis of our objective to determine how one player adapts to the other. To this end, we resorted to an incremental task of uncovering tiles one by one. Moreover, earlier pilot runs showed that a smaller grid didn’t invite players to divide it between them, and that a more demanding task was more likely to be divided. We adapted the second to last paragraph of ‘The task’ section to address this issue, and the reviewer’s query as to why we chose the grid size that we did.
- The reviewer is concerned that, in our task, the need for iterations to achieve coordination may be “an artefact of the complexity of the task and the number of different focal points available”. After careful consideration we think that this is not a problem, but an important claim we want to make. Schelling’s original examples were all one-shot tasks, featuring complex problems (e.g., if you and your wife get lost from one another in the supermarket, in the era prior to cell phones, where will you find each other?) but the focal point solution only works if there is just one focal point (can you and your wife think of only one single salient spot in the supermarket?). The fact is that two or more focal points call for further negotiations. It is no surprise that solving a task with two or more focal points, just as our task, requires iterations to achieve coordination. We don’t see this as a shortcoming, but rather addressing the issue at hand, namely, how is this implicit negotiation achieved? We modified the fifth paragraph in the ‘Results’ section where this issue is brought up and included a paragraph in the ‘Discussion’ section to make explicit that multiple focal points require further negotiations and how our proposal addresses this issue.
- We added to the ‘Discussion’ section some suggestions about how our findings are applicable to multi-agent systems and human-robot collaboration.
- We fixed the second sentence of the introduction to avoid repeating ourselves.
- In the third paragraph of the ‘Introduction’ section, we have included an example of a (partial) payoff matrix to make the presentation easier to follow.
- We have provided in the ‘The task’ section a number of real-world situations that can be modelled by our task to better motivate it. The generalization to an n-person interaction task known as the `El Farol bar problem’ is mentioned in the ‘Discussion’ section as a possible application of our FRA model.
