# Supplementary material for: Self-organized division of cognitive labor
Source: PLoS One. 2021 Jul 19;16(7):e0254532. doi: 10.1371/journal.pone.0254532 (PMC8289079; doi:10.1371/journal.pone.0254532)
Supplement: Attachment — Submitted filename: Response to Reviewers.docx [file pone.0254532.s002.docx]

**Response to Reviewers**

Response to reviewer 1’s comments:

- The reviewer was concerned about the typos in the text. We made a thorough review to fix as many of them as possible.
- In the previous versions of the manuscript, we used a linear regression to explore the relationship between score on round n-1 and consistency on round n. The reviewer was concerned that this was not an adequate model of the data and suggested we should use an ordinal logistic regression instead. To implement this analysis, we partitioned both variables into three intervals, so that the analysis could be carried out on two ordinal variables. We explored the relationship between the levels of these variables by means of an ordinal logistic regression. We showed that the results favor a positive relationship from low scores to high scores suggesting that trials where players received a high score elicited a more consistent behavior on the next round as compared to trials where players received low or medium scores. We visualized this relationship by means of a comparison of the boxplots of consistency for the three levels of score. This plot now replaces the original linear regression in Figure 5. A similar analysis was conducted with the simulated data and the regression in Figure 7.
- We added an extra section at the end of the manuscript in which we groupe the explanation of all our data and code. We provided a brief explanation of each resource and provided links to their respective repositories.
- Our datasets are improved with a detailed explanation in a README file (as well as a jupyter notebook) and are located not only in the main Github repository, but also in the Open Science Foundation website (https://osf.io). Links to the datasets and explanations are added to the “Data and resources availability” section. We decided against the Datadryad website to store our datasets because at this moment we cannot afford their fee.
